# Transplanted Neural Stem Cells: Playing a Neuroprotective Role by Ceruloplasmin in the Substantia Nigra of PD Model Rats?

**DOI:** 10.1155/2015/618631

**Published:** 2015-06-03

**Authors:** Jia-Jia Xiao, Ming Yin, Ze-Jian Wang, Xiao-Ping Wang

**Affiliations:** ^1^Department of Neurology, Shanghai First People's Hospital, School of Medicine, Shanghai Jiao Tong University, Shanghai 200080, China; ^2^School of Pharmacy, Shanghai Jiao Tong University, Shanghai 200240, China

## Abstract

Although mounting evidence suggests that ceruloplasmin (CP) deficiency and iron deposition are pivotal factors responsible for exacerbating demise of dopaminergic neurons in the substantia nigra (SN) of the Parkinsonism and neural stem cells (NSCs) are believed to be excellent candidates for compensating the lost dopaminergic neurons, there are few researches to explore the change of CP expression and of iron deposition in the pathological microenvironment of SN after NSCs transplantation and the ability of grafted NSCs to differentiate directionally into dopaminergic neurons under the changed homeostasis. With substantia nigral stereotaxic technique and NSCs transplantation, we found that tyrosine hydroxylase and CP expression decreased and iron deposition increased in the lesioned SN after 6-OHDA administration compared with control, while tyrosine hydroxylase and CP expression increased and iron deposition decreased after NSCs transplantation compared to 6-OHDA administration alone. Only a small number of embedding NSCs are able to differentiate into dopaminergic neurons. These results suggest that grafted NSCs have an influence on improving the content of CP expression, which may play a neuroprotective role by decreasing iron deposition and ameliorating damage of dopaminergic neurons and possibly underline the iron-related common mechanism of Parkinson's disease and Wilson's disease.

## 1. Introduction

Neural stem cells (NSCs) are renewable cells, famous for the specialties of self-replication and multilineage differentiation, making it possible to compensate the lost neurocytes and improve the symptoms of neurodegenerative diseases such as Parkinson's disease (PD) [1]. Exogenous transplantation is a promising strategy for rebuilding dopaminergic circuits and postponing process in PD [2]. In relevant animal models, NSCs transplantation has succeeded in neuroprotective effects against dopaminergic damage [3, 4]. Previous studies reported that NSCs were able to migrate directly towards damaged substantia nigra (SN) rather than other brain regions, disperse throughout the substantia nigra pars compacta, and develop partly neonatal dopaminergic neurons which are helpful to dopaminergic reinnervation [5–7]. The levels of gene expression of multifarious neural trophic factors such as nerve growth factor (NGF), brain-derived neurotropic factor (BDNF), and neurotrophin-3 (NT-3) increase significantly* in vitro* NSCs culture and* in vivo* NSCs transplantation; these trophic factors can not only promote grafted NSCs to better adapt to ischemia microenvironment and facilitate homeostasis in brain, but also protect remaining endogenous brain cells against further damage [8, 9]. NSCs proliferation and neural plasticity are seemingly associated with the progress of the disease and the severity of pathology [10, 11]. However, it is more complicated for the pathogenesis and homeostasis in PD patients compared to animal models. There is a necessity to focus on the more efficient differentiation of NSCs towards dopaminergic neurons. But, at the same time, the changes of the pathologic microenvironment about the homeostasis of ceruloplasmin (CP) and iron should not be neglected, which may impact on NSCs function and PD symptoms after NSCs transplantation.

As a multicopper ferroxidase, CP is mainly expressed by astrocytes in the brain and anchored in the cytomembrane surface, involving iron metabolism and copper homeostasis both in PD and in Wilson's disease (WD) [12, 13]. PD is considered to be closely related to the degraded ferroxidase activity and declined antioxidant defenses [14]. In the SN, a deficiency of ferroxidase activity of CP contributes to the prooxidant ferrous iron accumulation and Parkinsonian neurodegeneration, resulting in 30% nigral loss in CP knockout mice [15]. Aceruloplasminemia (aCP) [16] is characteristic with CP deficiency and iron deposition in central nervous system and peripheral tissue, causing diversified pathological alterations such as parkinsonism, craniofacial dyskinesia, cognitive impairment, and cerebellar ataxia, which is often present both in PD and in WD [10, 17]. Parkinsonism and iron concentration are attenuated by iron chelation with deferiprone in CP^−/−^ mice and aCP [16, 18]. However, little information is available about the pathophysiological changes of iron and CP accompanying NSCs transplantation. Furthermore, the mechanism of NSCs differentiated into tyrosine hydroxylase-immunoreactive (TH-ir) neurons is still ambiguous [19]. Inconsistent results have been illustrated among different researches and this subject remains controversial [11, 20]. The purpose of this paper is to evaluate the levels of iron, CP, and TH-ir neurons after NSCs transplantation in the SN of PD, which may offer theoretical support for replacement therapy with NSCs.

## 2. Materials and Methods

### 2.1. Animal Models

Female Sprague-Dawley rats (Slac Laboratory Animal Technology Co., Ltd.) weighing 180–220 g were housed under the circumstance of room temperature, constant airflow, acoustic isolation, and 12 h light/dark cycles, with ad libitum food and water. All the animals were randomly split into three groups: control, 6-OHDA-treated, and NSC-grafted 6-OHDA-treated groups. Rats in 6-OHDA-treated and NSC-grafted 6-OHDA-treated groups were anesthetized with chloral hydrate (400 mg/kg, i.p.) and immobilized to the stereotaxic apparatus. A small burr hole was drilled into the skull above the right medial forebrain bundle (MFB) region and then 6-OHDA (2 *μ*g/*μ*L, Sigma) was microinjected (8 *μ*L) at the rate of 0.5 *μ*L/min into the right MFB using stereotaxic technique in accordance with two coordinates: anteroposterior (AP): −1.8 mm, mediolateral (ML): 2.5 mm, and dorsoventral (DV): −7.5 mm and AP: −1.8 mm, ML: 2.5 mm, and DV: −8.0 mm from bregma [21–23]. After microinjection, the needle was left in coordinate site for 5 min before being removed slowly. Healthy rats without any process were selected as control. All the rats received rotational tests by apomorphine hydrochloride (0.05 mg/kg, s.c., Sigma) one week before and four weeks after surgery. Asymmetrical rotations were counted within 30 min and reached more than 7 rotations/min; they were deemed to be PD model rats [24]. Each group was of fifteen rats: five rats were used for immunohistochemical staining, five for Western blot, and also five for real-time PCR. All researches were conducted strictly according to the Guide for the Care and Use of Laboratory Animals (NIH) and approved by the Animal Ethics Committee of Shanghai Jiao Tong University.

### 2.2. NSCs Transplantation

NSCs were acquired from the subventricular zone (SVZ) of E14 fetal rat brains. The dissociated tissue was digested with 0.25% Trypsin-EDTA and the cell suspension was then incubated in serum-free medium (DMEM/F12, Hyclone) containing epidermal growth factor (EGF, 20 ng/mL, Invitrogen), basic fibroblast growth factor (bFGF, 20 ng/mL, Invitrogen), and B27 supplement (20 *μ*L/mL, Invitrogen). Four weeks after 6-OHDA lesion, PD rats were injected 5 *μ*L NSCs suspension (5 × 10^4^ cells/*μ*L) into the right SN as the following coordinate: AP: −5.2 mm, ML: 1.8 mm, and DV: −7.8 mm [6]. The speed was 0.5 *μ*L/min and needle retaining time was 10 min.

### 2.3. Histological Studies

All the animals were killed over eight weeks after the last treatment. Rats were fully anesthetized and perfused transcardially with 200 mL of cold PBS, followed by 300 mL 4% paraformaldehyde. The harvested brains were immersed in the 4% paraformaldehyde (4°C) for fixation and subsequently soaked in 20% and 30% sucrose solutions (4°C) for dehydration. The coronal sections of SN were cut into 10 *μ*m on a freezing microtome and stored at −20°C.

### 2.4. Iron Staining

Iron staining was on the basis of Perls' Prussian blue reaction: the sections were washed for three times with 0.01 M phosphate-buffered saline (PBS) and incubated for 30 min at 37°C in the mixed solution of equal parts, 2% HCl and 2% potassium ferrocyanide freshly prepared. By washing three times with PBS, sections were soaked in 99.7% methanol containing 0.3% hydrogen peroxide for 20 min in order to inactivate endogenous peroxidases vitality. They were washed again and then coloured in the solution of diaminobenzidine (DAB) for 10 min.

### 2.5. Immunohistochemistry

The sections were washed three times by 0.01 M PBS and treated with 0.3% hydrogen peroxide methanol solution for 20 min to suppress endogenous peroxidases activity. After washing thrice in PBS, these sections were treated for 30 min at room temperature with 5% bovine serum albumin for the purpose of reducing nonspecific binding and then incubated overnight with rabbit anti-rat TH (1 : 1,000, GeneTex) [27] and rabbit anti-rat CP (1 : 1,000, Epitomics) [28] at 4°C in a humidified chamber. The sections were washed with PBS and incubated with biotinylated goat anti-rabbit IgG (Boster) for 30 min at 37°C, accompanied with enlargement through streptavidin peroxidase for 30 min at the same temperature. Afterwards, sections were rinsed by PBS and coloured with DAB kits for 10 min.

### 2.6. Neuronal Counting

Sections were counted manually by a light microscopy with ×400 magnification and three images per section were taken to calculate positive neurons at an average. The counting areas were mainly located in the central substantia nigra pars compacta (SNpc) region. All counts and assessments were carried out blindly to the treatments received.

### 2.7. Western Blot

Fresh right nigral tissues were dissected out, respectively, from 6-OHDA-treated, NSC-grafted 6-OHDA-treated and control groups, homogenized in cold lysis buffer supplemented with 1 M Tris-HCl, 1% nonidet p-40, 1% Triton X-100, 0.5% sodium deoxycholate, 10% SDS, 0.5 M EDTA, 1 mM PMSF, and protease inhibitor cocktail (10 *μ*g/mL aprotinin, 10 *μ*g/mL leupeptin), and then centrifuged at 12000 ×g for 10 min at 4°C to collect the supernatant. Protein concentrations were assessed by the Bradford protein assay. Equal amounts of protein were diluted in loading buffer and then separated by SDS-PAGE (10% gradient gels) and transferred to nitrocellulose membrane (CP: 120 V, 120 min; TH: 60 V, 90 min). After transfer, samples were washed three times with TBST for 5 min per time and blocked by 5% skim milk-TBST buffer for 2 h at room temperature. The membranes were incubated at 4°C overnight with primary antibodies, anti-CP (1 : 2000, Epitomics) and anti-TH (1 : 2000, GeneTex), and then incubated with the secondary antibody (1 : 20000, Boster) for 1 hour at room temperature. Target proteins were visualized by using enhanced chemiluminescence reagents. Densitometry analysis was applied to assess semiquantifications of the signals with the MetaMorph software.

### 2.8. Real-Time Quantitative PCR

Total RNA was purified by homogenization in Trizol reagent from right nigral tissues. Extracted RNA (2 *μ*g) was transcribed reversely into cDNA using the AMV reverse transcription system. The primer sequences were shown in Table 1.

PCR amplifications were carried out as the following conditions: step 1, 5 min at 94°C for one cycle and step 2, 30 s at 94°C, 30 s at 57°C, and 1 min at 72°C, for 30 cycles [25]. The data were assessed by the delta-delta Ct method (2^−ΔΔCT^).

### 2.9. Statistical Analysis

SPSS 19.0 software was used to statistically analyze all data. The results were expressed exactly as the mean ± SD and assessed by one-way ANOVA followed by the SNK test. Probability *p* value of less than 0.05 was deemed to be statistically significant difference.

## 3. Results

### 3.1. Grafted NSCs Increased TH-ir Neurons in the SN of 6-OHDA-Induced PD Rats

**Figure 1:**
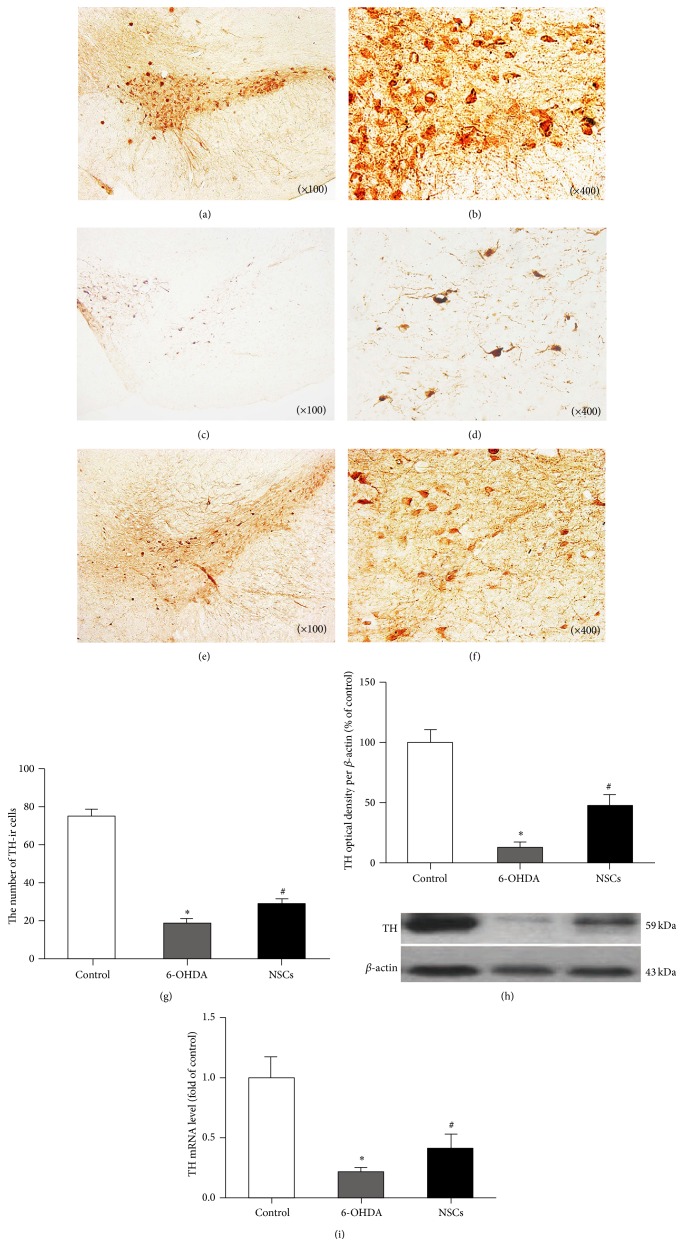
Influences of grafted NSCs on the TH-ir neurons in the SN of 6-OHDA-induced PD rats. (a, b) TH-ir neurons in control rats; (c, d) TH-ir cells in 6-OHDA-treated rats; (e, f) TH-ir neurons in NSCs-treated rats; (g, h, and i) the average total number of TH-ir neurons, Western blot, and real-time quantitative PCR in different groups: TH-ir neurons significantly decreased in the SN of 6-OHDA-treated rats compared with control rats (^*∗*^ *p* < 0.05), while they increased in NSCs-treated rats compared with 6-OHDA-treated rats (^#^ *p* < 0.05).

TH-ir neurons presented multiple dendrites, plump somata, and high density in the SN of normal rats (75.42 ± 11.24) (Figures 1(a) and 1(b)), while there were few dendrites, slight somata, and significantly decreased density of TH-ir neurons in the lesioned SN of 6-OHDA-treated (18.75 ± 8.98) (Figures 1(c) and 1(d)) and NSC-grafted 6-OHDA-treated (28.58 ± 9.17) (Figures 1(e) and 1(f)) groups. Cell counting (Figure 1(g)) indicated that the number of TH-ir neurons was, respectively, decreased by 75% and 62%. Compared with the 6-OHDA-treated rats, the density of TH-ir neurons in NSC-grafted 6-OHDA-treated groups was increased by 52% after grafting NSCs but still far from normal level. NSCs treatment induced a mild increase in TH mRNA expression and protein synthesis in lesioned SN (Figures 1(h) and 1(i)).

### 3.2. Transplanted NSCs Decreased Iron-Staining Cells in the SN of 6-OHDA-Induced PD Rats

**Figure 2:**
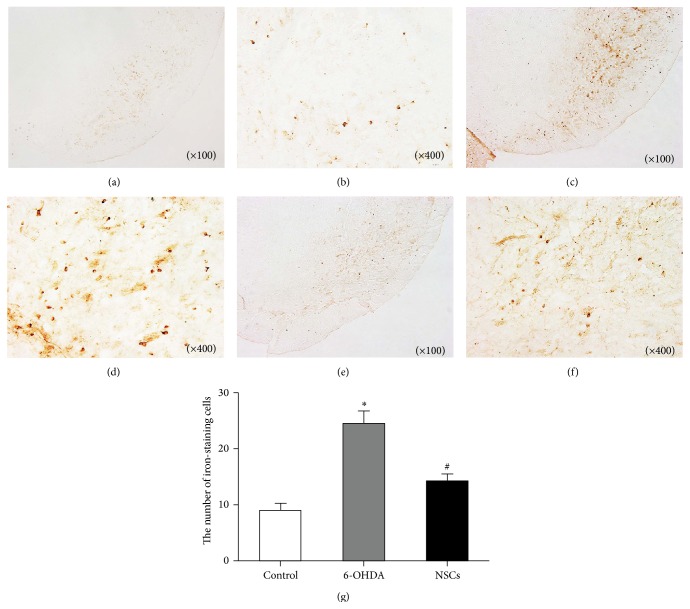
Effects of transplanted NSCs on the iron-staining cells in the SN of 6-OHDA-induced PD rats. (a, b) Iron-staining cells in control rats; (c, d) iron-staining cells in 6-OHDA-treated rats; (e, f) iron-staining cells in NSCs-treated rats; (g) the average total number of iron-staining cells in different groups: iron-staining cells dramatically increased in the SN of 6-OHDA-treated rats compared with control rats (^*∗*^ *p* < 0.05), while they decreased in NSCs-treated rats compared with 6-OHDA-treated rats (^#^ *p* < 0.05).

In the SN of healthy rats, a very small quantity of iron-staining cells (8.83 ± 5.13) (Figures 2(a) and 2(b)) was observed. But in the lesioned SN of 6-OHDA-treated groups, the positive number of iron-staining cells was dramatically increased by 178% (24.54 ± 7.86) (Figures 2(c) and 2(d)); the staining density of the lesioned to the healthy side was obviously darkened and turbid. After transplanting NSCs, the positive cells amount was decreased by 42% (14.17 ± 4.75) (Figures 2(e) and 2(f)).

### 3.3. Embedding NSCs Enhanced CP Expression in the SN of 6-OHDA-Induced PD Rats

**Figure 3:**
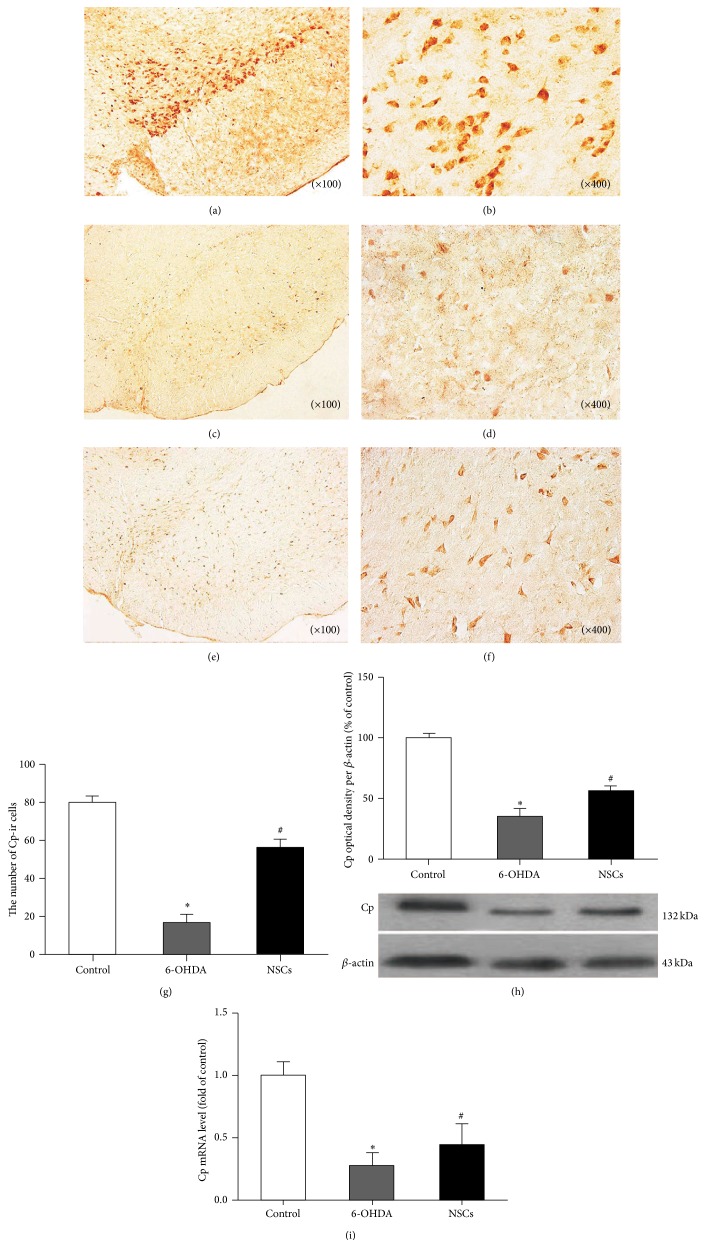
Impacts of exogenous NSCs on the Cp-ir cells in the SN of 6-OHDA-induced PD rats. (a, b) Cp-ir cells in control rats; (c, d) Cp-ir cells in 6-OHDA-treated rats; (e, f) Cp-ir cells in NSCs-treated rats; (g, h, and i) the average total number of Cp-ir cells, Western blot, and real-time quantitative PCR in different groups: Cp-ir cells significantly decreased in the SN of 6-OHDA-treated rats compared with control rats (^*∗*^ *p* < 0.05), while they increased in NSCs-treated rats compared with 6-OHDA-treated rats (^#^ *p* < 0.05).

There were massive CP-immunoreactive (CP-ir) cells (79.83 ± 12.19) (Figures 3(a) and 3(b)) in the physiological microenvironment of SN in control group. However, the quantity of CP-ir cells in surgery groups was diminished especially in 6-OHDA-treated groups. Staining results showed that the density of CP-ir cells in the lesioned SN of 6-OHDA-treated and NSC-grafted 6-OHDA-treated groups was, respectively, decreased by 78% (17.25 ± 11.34) (Figures 3(c) and 3(d)) and 30% (56.13 ± 13.20) (Figures 3(e) and 3(f)) compared to control groups. Notably, exogenous NSCs enhanced CP expression in mRNA and protein level (Figures 3(h) and 3(i)) and also increased CP-ir cells (Figure 3(g)).

## 4. Discussion

TH-ir neurons are well-suited for assessing the loss of dopaminergic cells as well as the differentiation of NSCs in PD. In our study, grafted NSCs have also exerted protection on the remaining dopaminergic neuron and impact on proliferation in the 6-OHDA-induced rats. After injecting NSCs, the number of TH-ir neurons increased slightly but did not reach the physiological amounts. As was reported, more than 50% of the newly generated neurons will confront apoptosis and approximately 5–10% of the grafted NSCs expressed TH-ir neurons [29, 30]. Numerous lines of evidence have demonstrated that TH-ir neurons are significantly lessened in the SN of Parkinson's rats [15, 31] and exogenous NSCs transplanted into SN are able to survive, migrate, and also differentiate [11]. Nevertheless, in the midbrain of PD, the low oxygen tension, through upregulating hypoxia inducible factor-1 (HIF-1), accelerates NSCs proliferation, stimulates NSCs that differentiate into neonatal dopaminergic neurons which have been verified to secrete dopamine, and promotes erythropoietin (EPO) and vascular endothelial growth factor (VEGF) expression to further exert neuroprotective effects on midbrain dopaminergic neurons [32]. Moreover, reactive astrocytes support TH-ir neurons development and survival [33], and transplanted NSCs tend to differentiate into more astrocytes than dopaminergic neurons in our study. Interestingly, undifferentiated neural progenitor cells (NPCs) from mouse embryos embedded into the lesioned mouse striatum preferentially differentiate into neurons but not glial cells [34]. Perhaps such discrepancies are caused by various origins of grafted cells or different conditions of cell culture.

As a high activity of electron transfer, ferrous iron is supposed to be a forceful catalyst for the production of reactive oxygen species (ROS) by Fenton reaction; its accumulation will injure dopaminergic cytomembrane and chondriosome; as a result, dopaminergic neurons are degenerated in a large amount in PD [35]. Iron-staining cells are the damaged neurons and gliacytes, but dopaminergic neurons are more vulnerable. Supporting this notion is the fact that SN in the brain is the second highest concentration of iron behind globus pallidus [36], in which iron progressively deposits with aging process [37], coincident with areas more susceptible to insults. The oxidases such as glutathione peroxidase (GSH) and superoxide dismutase (SOD) have a considerable shortage in dopaminergic neurons of SN compared to other regions of the brain [38, 39]. The lack of ferritin and neuromelanin digested by microglia in the dopaminergic neurons leads to the fact that intracellular iron exists mainly in the form of free ferrous iron [40]. What is more, high amounts of iron released from damaged neurogliocytes such as astrocytes and microglia promote dopaminergic neurons uptake [41]. Analogously, iron also accumulates in basal ganglia occurring in the whole pathological process of WD and induces secondary damage to affected tissues, although the deposition of copper has been verified to be the main pathogenic factors [42]. In the SN of Parkinson's rats, there are fewer TH-ir neurons but more iron-staining cells. In our study, we further observed that the density of iron-staining cells declined slightly in the lesioned SN after NSCs transplantation, suggesting that grafted NSCs might relieve iron deposition in dopaminergic neurons by enhancing CP content and activity.

Our study also showed that CP levels in the SN of NSC-grafted 6-OHDA-treated PD rats were predominantly higher than PD rats. Intracellular iron decrease was concurrent with increased levels of CP mRNA and protein. These phenomena suggest that transplanted NSCs are very likely to play a neuroprotective role by increasing the expression level of Cp, which may decline the deposition of intracellular iron by increasing the expression of ferroportin in the membrane surface of dopaminergic neurons or by decreasing ferrous iron into the cells. The synthesis of CP in the SN plays a crucial role in eliminating ferrous iron and protecting against iron-mediated free-radical injury towards TH-ir neurons [43]. High activity of CP promotes stable expression of ferroportin (Fpn), by which excessive iron will be exported from neuroglia cells and dopaminergic neurons to extracellular matrix [44]. Once the content and activity of CP decline, due to gene mutation, aging process, or toxic tort, ferrous iron will access cytomembrane conveniently by means of divalent metal transporter (DMT1). Similarly, CP-deficient mice are more vulnerable in oxidative stress rather than wild-type mice [45]. Besides, Fpn expressing in low level leads to decreased iron efflux, all of which aggravate cellular iron overload and oxidative damage [46].

In conclusion, NSCs are capable of differentiating into TH-ir neurons and ameliorating surrounding microenvironment in the SN of Parkinson's rats. But most of all, by increasing the number of CP-ir cells and improving the content of CP, exogenous NSCs transplantation declines the concentration of iron followed by relieving damage of dopaminergic neurons and may ameliorate the iron-related oxidative injuries of PD and WD.

## Figures and Tables

**Table 1:** 

Gene	Sequence (5′-3′)
CP [25]	Forward: GTA TGT GAT GGC TAT GGG CAA TGA
	Reverse: CCT GGA TGG AAC TGG TGA TGG A

TH [26]	Forward: AGC TGT GCA GCC CTA CCA AGA
	Reverse: GTG TGT ACG GGT CAA ACT TCA CAG A

*β*-actin	Forward: CCT CTA TGC CAA CAC AGT
	Reverse: AGC CAC CAA TCC ACA CAG
